# Exome Sequencing Identifies a Founder Frameshift Mutation in an Alternative Exon of *USH1C* as the Cause of Autosomal Recessive Retinitis Pigmentosa with Late-Onset Hearing Loss

**DOI:** 10.1371/journal.pone.0051566

**Published:** 2012-12-12

**Authors:** Samer Khateb, Lina Zelinger, Tamar Ben-Yosef, Saul Merin, Ornit Crystal-Shalit, Menachem Gross, Eyal Banin, Dror Sharon

**Affiliations:** 1 Department of Ophthalmology, Hadassah - Hebrew University Medical Center, Jerusalem, Israel; 2 Genetics Department, Rappaport Faculty of Medicine, Technion - Israel Institute of Technology, Haifa, Israel; 3 Department of Ophthalmology, Meir Medical Center, Kfar-Saba, Israel; 4 Department of Otolaryngology - Head and Neck Surgery, Hadassah - Hebrew University Medical Center, Jerusalem, Israel; Radboud University Nijmegen Medical Centre, The Netherlands

## Abstract

We used a combined approach of homozygosity mapping and whole exome sequencing (WES) to search for the genetic cause of autosomal recessive retinitis pigmentosa (arRP) in families of Yemenite Jewish origin. Homozygosity mapping of two arRP Yemenite Jewish families revealed a few homozygous regions. A subsequent WES analysis of the two index cases revealed a shared homozygous novel nucleotide deletion (c.1220delG) leading to a frameshift (p.Gly407Glufs*56) in an alternative exon (#15) of *USH1C*. Screening of additional Yemenite Jewish patients revealed a total of 16 homozygous RP patients (with a carrier frequency of 0.008 in controls). Funduscopic and electroretinography findings were within the spectrum of typical RP. While other *USH1C* mutations usually cause Usher type I (including RP, vestibular dysfunction and congenital deafness), audiometric screening of 10 patients who are homozygous for c.1220delG revealed that patients under 40 years of age had normal hearing while older patients showed mild to severe high tone sensorineural hearing loss. This is the first report of a mutation in a known USH1 gene that causes late onset rather than congenital sensorineural hearing loss. The c.1220delG mutation of *USH1C* accounts for 23% of RP among Yemenite Jewish patients in our cohort.

## Introduction

Retinitis pigmentosa (RP) [MIM #268000] is the most common inherited retinal degeneration with an estimated worldwide prevalence of about 1∶4000 [1], [2]. The disease is highly heterogeneous and can be inherited in autosomal dominant, autosomal recessive (AR), and X-linked modes. In most cases, RP is nonsyndromic (e.g. no other clinical features are evident), but in about 20–30% of cases the patients manifest a syndrome, such as Usher syndrome (USH) which also causes vestibular and hearing impairment to varying degrees, Bardet-Biedl syndrome (BBS), and others [3]. At present, 36 genes have been associated with arRP and 10 with USH (https://sph.uth.tmc.edu/RetNet/home.htm). An interesting group of retinal disease genes can cause both syndromic and nonsyndromic phenotypes, not always with a clear disease mechanism: for example, *USH2A* mutations can cause the USH phenotype but also RP alone [4], [5], [6], [7], [8]; *USH1C* and *MYO7A* mutations have been associated with the USH phenotype but also hearing impairment alone [9], [10].

The Yemenite Jewish (YJ) population is ancient (over 2,000 years) and comprised of several small communities. Most members of this population immigrated back to Israel between 1950 and 1955. In 2010, Israeli residents of YJ descent comprised about 2.5% (140,000) of the Israeli Jewish population. Up to date, a single founder mutation in the *CERKL* gene (c.238+1G>A) has been reported by us as the cause of inherited widespread retinal degeneration with early macular involvement in about 30% of cases of YJ origin [11].

In order to identify additional causes of RP in this population, we recruited 36 families of YJ origin with isolate/AR retinal degenerations (33 with RP and three with cone-rod degeneration). The set of RP families of YJ origin comprises 16% of our cohort of Israeli Jewish patients (33/205) which is much higher than the fraction of YJ among all Israeli Jews. This difference can by explained by founder mutations that are relatively common among YJ, causing higher prevalence of disease. We identified the *CERKL* founder mutation (c.238+1G>A) in 13 of the YJ families. We performed homozygosity mapping using whole-genome single nucleotide polymorphism (SNP) arrays in six patients who belong to two YJ families followed by whole exome sequencing (WES) analysis. Using this method we identified a novel frameshift mutation in an alternative exon of *USH1C* as the cause of RP with late onset hearing loss rather than the classic USH1 phenotype which manifests with profound congenital hearing loss and vestibular dysfunction.

## Materials and Methods

### Subjects and Clinical Evaluation

The tenets of the Declaration of Helsinki were followed and prior to donation of a blood sample, a written informed consent was obtained from all individuals who participated in this study, after explanation of the nature and possible consequences of the study. The research was approved by the institutional review board (IRB) at the Hadassah medical center. DNA was extracted from the index patient as well as from other affected and unaffected family members using the FlexiGene DNA kit (QIAGEN).

**Figure 1:**
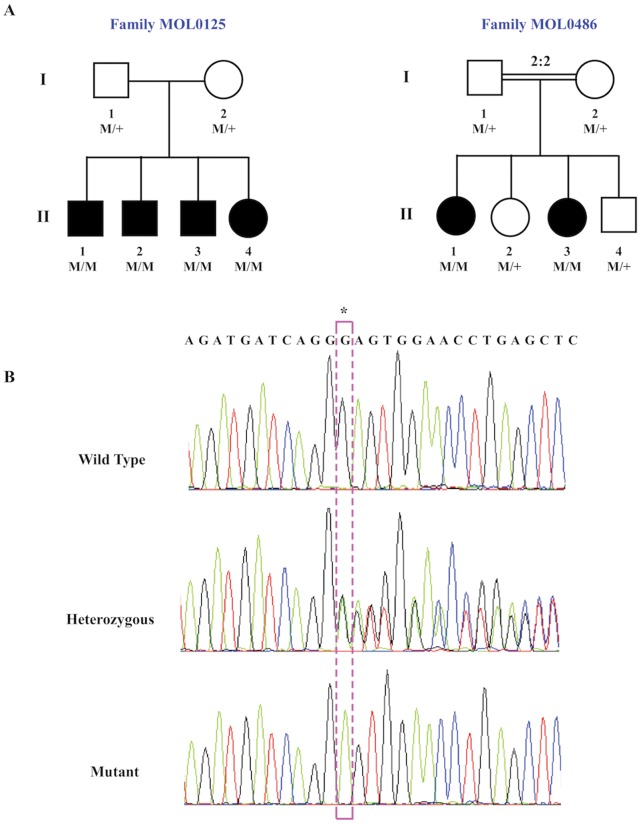
Family trees and segregation analysis of the *USH1C* c.1220delG mutation. A. Structure of the two families that were selected for whole exome sequencing. The family number is indicated above the corresponding family tree. Filled symbols represent affected individuals, whereas clear symbols represent unaffected individuals. Generation numbers are depicted on the left and individual numbers below each symbol. The *USH1C* genotype is presented as: M/M, homozygous for the c.1220delG mutation; M/+, heterozygous; +/+, homozygous for the wildtype allele. Index cases are marked with an arrow. B. Sequence chromatograms of part of *USH1C* exon 15 of a control individual (top), a heterozygote (middle), and a patient who is homozygous for the mutation (bottom). The c.1220delG mutation (boxed) results in a frameshift (p.Gly407Glufs*56).

Ocular evaluation included a full ophthalmologic exam, Goldmann perimetry, electroretinography (ERG), color vision testing using the Ishihara 38-panel and Farnsworth-Munsell D-15 tests, color and infrared fundus photos, optical coherence tomography (OCT), and fundus autofluorescence (FAF) imaging performed as previously described [12].

**Figure 2:**
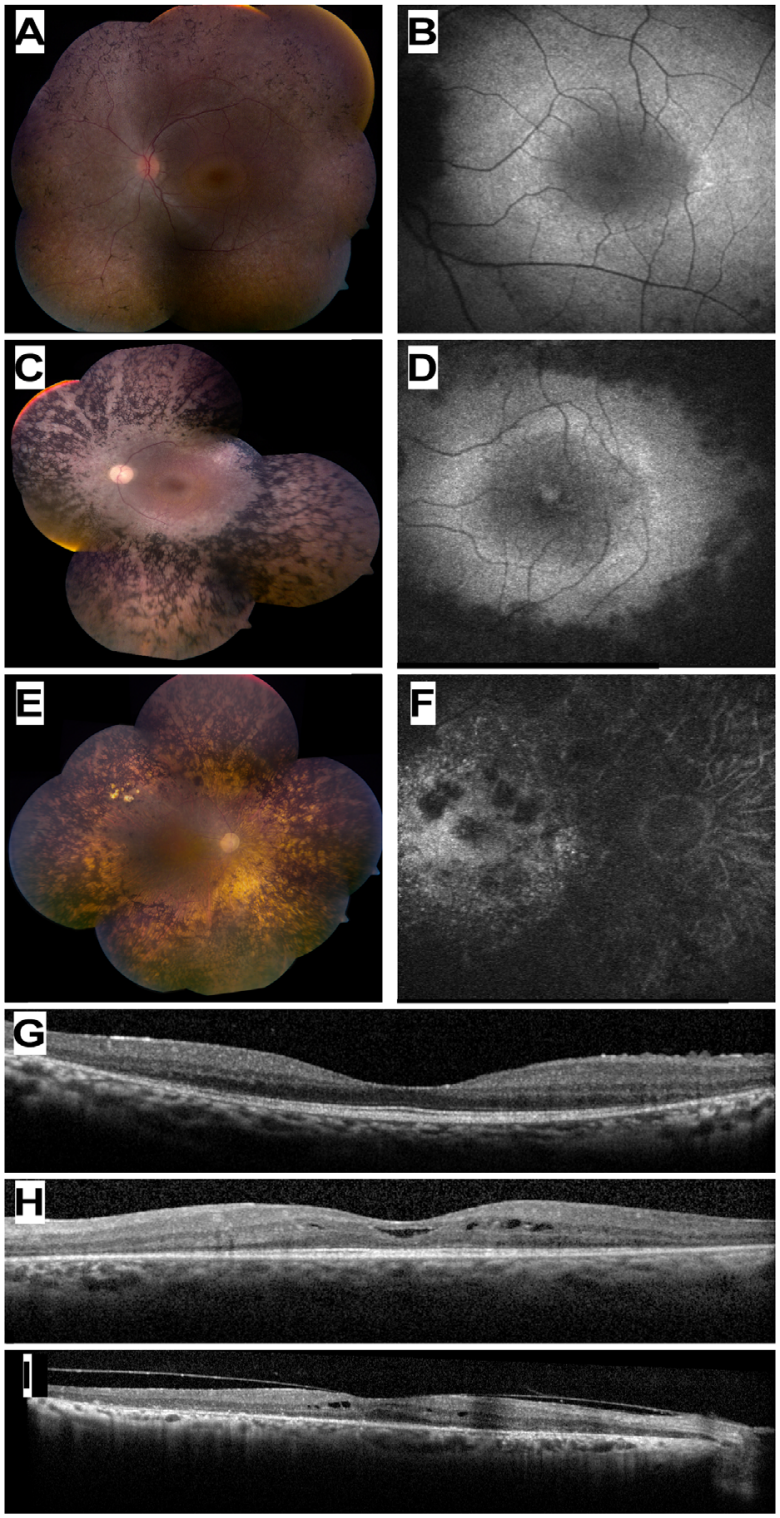
Ocular Phenotype of patients who are homozygous for the *USH1C* c.1220delG mutation. (A, C, E) Color fundus photographs of three patients aged 13 (MOL0486 II:3), 33 (TB16-R12), and 72 (MOL1023-1) years old, respectively. Note the increasing severity of fundus changes with age, and the presence of dense bone spicule-like pigmentation. (B, D, F) Corresponding fundus autofluorescence imaging of the macular area in the three patients shown in A,C,E. Note hyeprfluorescent rings around the foveas in the younger patients, and hypofluorescent areas of atrophy that encroach upon the macula in the 33 year-old patient and invade the macula in the 72 year-old. (G, H, I) Horizontal optical coherence tomography (OCT) cross-sections through the fovea in the 13 yo (panel G), 33 yo (H) and 72 yo (I) *USH1C* patients showing progressive loss of retinal and particularly photoreceptor layer thickness with age. Intra-retinal cysts of fluid (cystoid macular edema) are evident in two of the cases (H, I).

Audiological screening included a comprehensive questionnaire (collecting information regarding any history of exposure to noise, ototoxic agents, and genetic factors related to hearing impairment), as well as physical and audiometric examinations. An age-appropriate audiological examination was performed including pure-tone audiometry (250 Hz to 12,000 Hz), tympanometry, and transient-evoked otoacoustic emission (TEOAE) for each ear. We used the following scale to grade the degree of sensorineural hearing loss (SNHL): slight- 16 to 25 dB hearing loss, mild- 26–40 dB, moderate- 41–55 dB, moderately severe- 56–70 dB, severe- 71–90 dB, and profound- >90 dB hearing loss [13]. Specific types of SNHL were determined by audiometric curve patterns: ascending (hearing loss greater at the lower frequencies), flat, descending (hearing loss greater at the higher frequencies), and "U-shaped" (hearing loss at mid-frequencies) curves. Otoacoustic emissions (OAEs) have been suggested as a sensitive measure of cochlear function with the potential for preclinical detection of damage [14]. TEOAE tests were conducted on the same day as the pure tone test. A response at three frequencies of 3 dB or greater above the background with a minimum of 70% reproducibility at each frequency and 90% or greater stability was required for passing the TEOAE test. TEOAE examination was categorized as either pass or fail for each ear.

**Figure 3:**
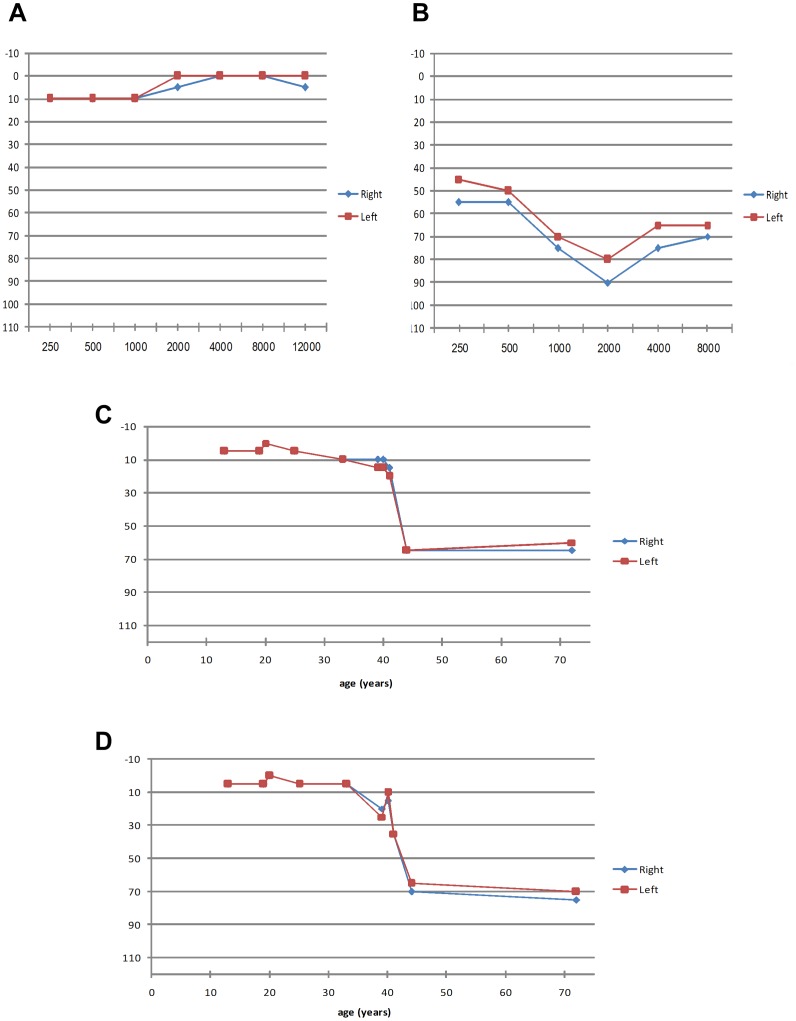
Audiometric results of patients who are homozygous for the *USH1C* c.1220delG mutation. A. Patient MOL0486 II:3 (13 yo) with normal hearing function. B. Patient MOL0887-3 (44 yo) with severe SNHL, particularly at higher frequencies (descending pattern). C,D. Hearing threshold versus age at a frequency of 4,000 Hz (C) and 8,000 Hz (D). At younger ages, normal hearing function is evident, but after the age of 40 years, sensitivity is markedly reduced. For each of the 10 patients, the data from both the right and left ears is presented, showing symmetry between both ears.

### Genetic Analyses

Whole genome SNP analysis was performed on six patients with nonsyndromic arRP who belong to two different YJ families, using either the Affymetrix 10K or 6.0 microarrays and data analysis was performed using HomozygosityMapper (http://www.homozygositymapper.org/). WES analysis was performed at Otogenetics corporation using Roche NimbleGen V2 (44.1 Mbp) paired-end sample preparation kit and Illumina HiSeq2000 at a 31x coverage. Sequence reads were aligned to the human genome reference sequence (build hg19) and variants were called and annotated using the DNAnexus software package. Dataset files including the annotated information were analyzed using ANNOVAR according to the dbSNP database (build 135) with the following filtering steps: autosomal recessive inheritance; variant type including missense, nonsense, and splice-site; not within segmental duplications; minor allele frequency (MAF) less than 0.5%; SIFT score <0.05 when available; PolyPhen2 score >0.85 when available. Primers for *USH1C* exon 15 (Table S1) were designed using the Primer3 software [15] and Sanger sequencing of PCR products was used to verify the mutation and to screen additional patients and controls. Retinal RNA was isolated from human retina using TRI-reagent (Sigma-Aldrich) and RNA derived from different human tissues was purchased (Clontech; cat. #636643, lot No. 8101369A). cDNA was synthesized using the Verso cDNA kit (Thermo) in accordance with the manufacturer's protocol. PCR specific primers (Table S1) were designed with Primer3 to amplify two parts of the *USH1C* mRNA: exons 14 through 16 and exons 14 through 23. RT-PCR of a *PGM1* fragment was used as a control (Forward: TGGTGCTCTGGACCGGGTGG; Reverse: GCACTCCCAGTGCCGCTCAG).

**Figure 4:**
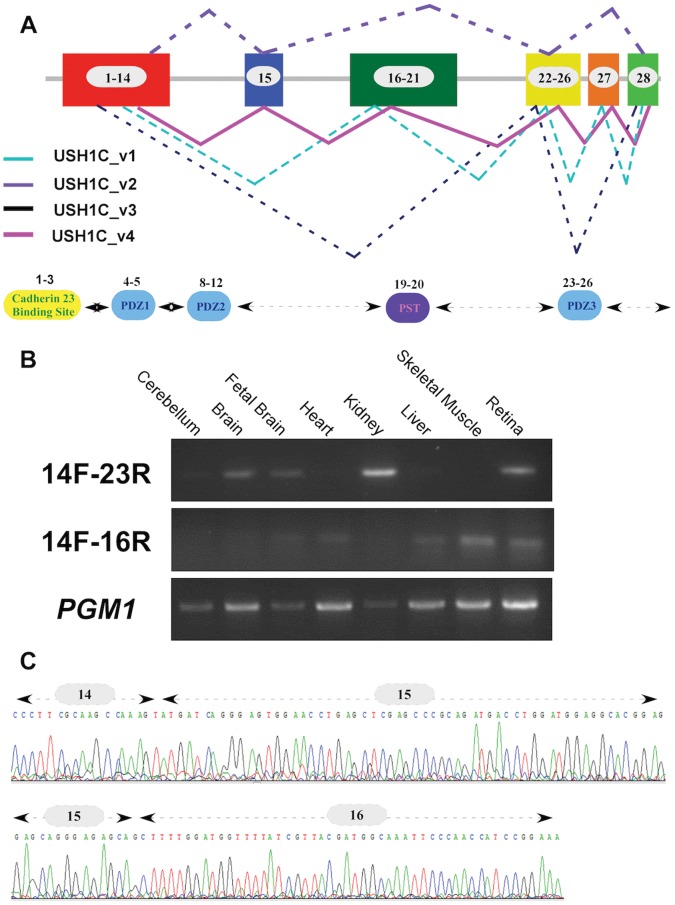
*USH1C* alternatively-spliced transcripts. A. *USH1C* can produce four different transcripts through alternative splicing. The scheme represents the full-length open reading frame (ORF) of *USH1C* and the alternatively-spliced transcripts that can be produced. Exon numbers are depicted within boxes. Previously reported transcripts are dashed and the novel transcript (USH1C_v4) is presented with a solid line. The bottom panel shows the USH1C functional domains and the corresponding coding exons. B. RT-PCR analysis of *USH1C* splicing variants. A PCR product of 249 bp (top panel) amplified by the 14F and 23R primers (Table S1), representing the most common *USH1C* splicing isoform, USH1C_v2, is depicted in different human tissues. The newly discovered transcript (USH1C_v4) is depicted in the middle panel (a PCR band of 189 bp amplified by primers 14F and 16R; Table S1) showing a relatively low expression level. Both PCR products were sequenced and verified in a few tissues, including the retina. *PGM1* RT-PCR was used as a control (bottom panel). C. Sequence chromatogram of the newly-identified *USH1C* splicing isoform. The splicing variant (USH1C_v4) contains all *USH1C* coding exons and the new splicing event (exon14-exon15-exon16) is depicted.

## Results

### Whole Genome SNP Analysis

We performed homozygosity mapping using whole genome SNP arrays in six patients who belong to two YJ families. Family MOL0125 is non-consanguineous with four affected siblings and family MOL0486 is consanguineous (the parents are first cousins) with two affected sisters (Fig. 1A). The index case of MOL0125 was screened for mutations using the APEX-based Asper arRP array (an arrayed primer extension-based genotyping process, including most of the arRP mutations thus far reported) and was homozygote for the wild-type allele in all tested nucleotides. Homozygosity mapping in MOL0486 revealed 6 and 5 homozygous regions (>10 Mb) in the two affected sisters, MOL0486 II:1 and MOL0486 II:3, respectively. Two of these regions were shared between the two affected sisters, the largest being a 38.98-Mb region on chromosome 11 between the markers rs1395558 and rs724759 (Table S2). A similar analysis of the four patients in MOL0125 did not reveal any large homozygous regions. The largest shared homozygous region was identified on chromosome 11 (Table S2) including 296 consecutive homozygous SNPs. This region was relatively small (about 1 Mb) including 9 genes, one of which is *USH1C*. We therefore could not exclude the possibility that the patients are compound heterozygous for two different mutations in the same gene.

### Whole Exome Analysis

Aiming to identify the genetic cause of disease in the two studied families, we performed WES analysis on one patient from each family (Table S3). For MOL0486 II:1, we produced 4.77 gigabases (Gb) of paired-end 100-nucleotide sequence reads, 88% of which were mapped to gene regions. A total of 933.93 million bases were covered with an average coverage of 30.17 reads per nucleotide (Table S3). Alignment of these reads to hg 19 revealed a total of 232,065 variants that underwent a series of filters (Table 1) to exclude sequence changes that are unlikely to be pathogenic. A total of 277 variants passed the filters, only one of which was within a genomic region that was homozygously shared by the two affected individuals in the whole genome SNP array analysis: a deletion of guanine (c.1220delG) leading to a frameshift (p.Gly407Glufs*56) in an alternative exon (#15) of *USH1C*.

**Table 1:** Data on sequence variants identified by the whole exome sequencing analysis.

Sample	Total no. ofvariants	No. of missense, nonsense and splicing variants	Variants in conserved regions and not in segmental duplications	Variants with MAF <0.5%	After exclusion of unlikely pathogenic missense changes	No. of variants fitting the disease model	No. of variants within linked regions
MOL0125 II:4	233,572	21,326	9,055	2,880	1,476	311	2*
MOL0486 II:1	232,065	20,469	8,245	2,536	1,252	277	2**

* - The two variants are: c.1220delG in *USH1C* and c.218_219insC in *VPS11*. The latter was found in about 73% of ethnicity-matched exomes and is therefore predicted to be a population-specific polymorphism.

** - The two variants are: c.1220delG in *USH1C* and c.705_706delTT in *PKD1L2*. The latter was found in about 20% of ethnicity-matched exomes and is therefore predicted to be a population-specific polymorphism.

In parallel, we performed a similar analysis of MOL0125 II:4. WES produced sequence reads in a total length of 4.51 Gb, 92% of the reads were mapped to gene regions with an average coverage of 31.17 reads per nucleotide (Table S3). The analysis revealed a total of 233,572 variants, only 311 remained (Table 1) after applying the same filters used in family MOL0486. As mentioned above, this family was nonconsanguineous and homozygosity mapping did not reveal large homozygous regions, we therefore considered both homozygous changes and two heterozygous changes affecting the same gene. The filtering based on disease model revealed only one possible mutation in *USH1C* as described above.

The combined whole genome SNP array and WES analysis in the two families revealed the same mutation- c.1220delG in *USH1C*. The *USH1C* mutation is part of a shared homozygous haplotype of 27 SNPs that is shared by the two index cases of families MOL0125 and MOL0486, indicating a founder mutation. We confirmed this mutation by Sanger sequencing (Fig. 1B) and it fully cosegregated with the phenotype in the two families (Fig. 1A).

### Mutation Analysis

Screening the c.1220delG mutation in a set of 119 ethnically-matched (YJ) normal controls revealed one heterozygous individual, thus indicating a carrier frequency of 0.008 (95% confidence interval 0.002–0.045) in this population. We subsequently screened a set of 35 unrelated YJ patients with retinal degenerations (21 of whom had nonsyndromic RP) for the c.1220delG mutation and identified six additional index RP cases who were homozygous for the mutation. Statistical analyses between patient and control groups showed a significant difference (Table S4).

### Clinical Evaluation

Aiming to clinically characterize patients with the c.1220delG mutation, we performed full ophthalmologic examinations and audiometric testing in most of the patients. Ocular evaluation of 12 patients showed a spectrum of findings (Table S5). Visual acuity ranged from 0.2 to 1.0, usually being better at younger ages. Funduscopic findings also showed worsening with age, including waxy-appearance of the optic nerve head, attenuation of retinal blood vessels, and increasing, prominent bone spicule-like pigmentation which already extended from the mid-periphery to encroach upon the posterior poles in patients in their twenties and early thirties (Fig. 2A, C, E). There was relative preservation of the macular area, but FAF imaging showed increased involvement with age (Fig. 2B, D, F). OCT imaging in early disease showed a relatively preserved photoreceptor layer in the foveal area, with a decline in outer nuclear layer (ONL) thickness with increasing distance from the fovea (Fig. 2G). In older patients, the ONL showed thinning also in the foveal area and cystoid macular edema was evident in some cases (Fig. 2H, I). ERG responses ranged from severely reduced to non-detectable in patients in their teens and twenties, and non-detectable at older ages (Table S5). Visual fields were markedly constricted in patients in their twenties (Fig. S1).

Audiometric testing revealed variable degrees of SNHL in four out of 10 examined patients, ranging from mild to severe symmetric hearing impairment (Table S6). Normal hearing was found in most participants under the age of 40 at all examined frequencies (250–12,000 Hz; see an example in Fig. 3A). Older patients did show SNHL, mainly at high frequencies (4,000–12,000 Hz). For example, patient MOL0887-3, at 44 years of age, showed moderate to severe hearing loss with a descending audiogram (Fig. 3B). An analysis of high-frequency thresholds versus age (Fig. 3C, D) showed normal hearing function in most patients (five out of six) below 40 years of age, while patients above 40 years showed higher tendency for SNHL (three out of four patients). Normal TEOAE responses were found in all participants who showed normal hearing on audiometry. All patients reported normal vestibular function.

### Expression Analysis

The human *USH1C* gene contains 28 coding exons along a genomic region of ∼37 Kb and is known to produce three splice variants [16] (variants USH1C_v1, USH1C_v2, and USH1C_v3; Table S7 and Fig. 4A). To characterize the expression of *USH1C* in human tissues, we analyzed all available *USH1C* EST sequences deposited at NCBI and noted three regions that are affected by alternative splicing: exon15, exons 16–21, and exon 27. Out of the 15 sequences encompassing exon 15, the vast majority (13 sequences) included exon 15 but not exons 21–26. Therefore, the most common *USH1C* variant is USH1C_v2 (also known as variant a and PDZ-73; see Table S7 for more information). RT-PCR analysis using a set of primers that are specific for this variant (primers 14F and 23R, see Table S1) revealed a variable expression level in most studied human tissues (Fig. 4B, top panel). Extensive RT-PCR analysis of this region revealed a novel splice-variant, USH1C_v4 (Table S7 and Fig. 4C) that includes all *USH1C* coding exons and is ubiquitously expressed at relatively low levels (Fig. 4B; middle panel using primers 14F and 16R, see Table S1).

## Discussion

We report here of the identification of a novel *USH1C* founder mutation in YJ patients with arRP. Some of the patients, mostly those above the age of 40 years, developed variable degrees of high tone SNHL. This *USH1C* mutation together with the previously reported *CERKL* founder mutation [11], account for about 50% of RP cases in this ethnic group.

*USH1C* encodes for harmonin, a scaffolding protein that is required for normal mechanosensory function in cochlear hair cells [17]. Its role in photoreceptors is not yet clear, although it may be involved in maintenance of the synaptic structure or in mediating the release of synaptic vesicles [18]. Mutations in *USH1C* were previously reported to cause the following phenotypes: Usher syndrome type 1 (characterized by congenital profound SNHL, vestibular dysfunction, and RP) [19], [20], nonsyndromic profound SNHL at the DFNB18 locus [9], [21], and sector RP with early-onset SNHL [22]. A complete list of mutations can be obtained from https://grenada.lumc.nl/LOVD2/Usher_montpellier/home.php?select_db=USH1C. Most patients with *USH1C* mutations suffer from typical USH1, usually due to null (including nonsense, frameshift, and splicing) mutations on both gene copies. The only exception is a patient who is a compound heterozygous for a null and a missense mutation but no clinical data were presented by the authors [23]. A few patients with less severe phenotypes (sector RP with early onset deafness and nonsyndromic profound deafness) carry mutation(s) that are expected to have a milder effect on protein amount or function. Two siblings who suffer from sector RP and hearing loss from the age of 4 years (but were able to develop language) were reported to be compound heterozygotes for a missense (p.Arg103His) and a canonical splice-site mutation (c.2227-1G>A) [22]. Nonsyndromic deafness was reported in only two families so far: patients from the original DFNB18 family were homozygous for a “leaky” splice-site mutation (IVS12+5G>C) that might affect the splicing of exons 11 and 12 [9], and patients from a Chinese family were homozygous for a missense change (p.Pro608Arg) but no functional assay was performed to prove the pathogenicity of this change [21]. Most of the mutations identified in *USH1C* so far (except for p.Pro608Arg and c.2227-1G>A) are located within the first 13 exons, which are present in all *USH1C* transcripts reported to date. The most common *USH1C* transcript, USH1C_v2, lacks exons 16–21 and exon 27, while two relatively rare variants (USH1C_v1 and USH1C_v3) lack exon 15. The founder mutation we report here causes a typical form of RP with prominent bone spicule-like pigmentation at relatively early ages, which is later complicated by late-onset SNHL. In the literature, the vast majority of *USH1C* patients were reported to manifest early and profound hearing loss, while the patients we report here maintain normal hearing until the age of about 40 years. This might be explained by partial compensation of harmonin function by the rare splicing variants that lack exon 15 (USH1C_v1 and USH1C_v3) and are therefore unlikely to be affected by the mutation. A relatively high expression or a more prominent role of these isoforms in the human cochlea versus the retina might explain the relatively mild and delayed hearing loss.

The data we report here together with previously reported studies suggest that mutations in *USH1C* may cause nonsyndromic hearing loss or RP (usually the typical form and rarely the sectorial form) with a variable effect on hearing (usually congenital profound deafness with vestibular dysfunction and rarely late-onset hearing loss). *USH1C* therefore should be considered a candidate gene in patients with USH1, nonsyndromic hearing loss, young (<40 years) RP patients with normal hearing function, and RP patients with mild or late-onset hearing loss. To the best of our knowledge, this is the first report showing that a mutation in a gene known to cause the USH1 phenotype can cause RP with late onset hearing loss.

## Supporting Information

Figure S1 **Goldmann perimetry of two patients who are homozygous for the** ***USH1C*** **c.1220delG mutation.** A. A 20 year-old patient (MOL0125 II:4) presented in 2007 with severely restricted visual fields which became worse 4 years later. B. The visual field of a 25 year-old patient (MOL0125 II:2) was markedly reduced in 2006 with additional progression 3 years later. The visual fields represent an average of both eyes.
(DOCX)

Table S1 **Primer sequences used to amplify the** ***USH1C*** **exon 15 from gDNA samples and the various transcripts of** ***USH1C.***
(DOCX)

Table S2 **Homozygosity mapping analysis of arRP families.**
(DOCX)

Table S3 **Summary of original exome sequencing data.**
(DOCX)

Table S4 **Statistical analysis of the c.1220delG mutation frequency.**
(DOCX)

Table S5 **Ocular data of patients with the c.1220delG** ***USH1C*** **gene mutation.**
(DOCX)

Table S6 **Hearing function tests: Audiometry and Transient Evoked Otoacoustic Emissions (TEOAEs).**
(DOCX)

Table S7 **Splice-variants produced by the human** ***USH1C*** **gene.**
(DOCX)

## References

[1] Haim M (2002) Epidemiology of retinitis pigmentosa in Denmark. Acta Ophthalmol Scand Suppl: 1–34. 10.1046/j.1395-3907.2002.00001.x 11921605

[2] Bunker CH, Berson EL, Bromley WC, Hayes RP, Roderick TH (1984) Prevalence of retinitis pigmentosa in Maine. Am J Ophthalmol 97: 357–365. 6702974 10.1016/0002-9394(84)90636-6

[3] Hartong DT, Berson EL, Dryja TP (2006) Retinitis pigmentosa. Lancet 368: 1795–1809. 17113430 10.1016/S0140-6736(06)69740-7

[4] Rivolta C, Sweklo EA, Berson EL, Dryja TP (2000) Missense Mutation in the USH2A Gene: Association with Recessive Retinitis Pigmentosa without Hearing Loss. Am J Hum Genet 66: 1975–1978. 10775529 10.1086/302926 PMC1378039

[5] Kaiserman N, Obolensky A, Banin E, Sharon D (2007) Novel USH2A mutations in Israeli patients with retinitis pigmentosa and Usher syndrome type 2. Arch Ophthalmol 125: 219–224. 17296898 10.1001/archopht.125.2.219

[6] Aller E, Najera C, Millan JM, Oltra JS, Perez-Garrigues H, et al (2004) Genetic analysis of 2299delG and C759F mutations (USH2A) in patients with visual and/or auditory impairments. Eur J Hum Genet 12: 407–410. 14970843 10.1038/sj.ejhg.5201138

[7] Xu W, Dai H, Lu T, Zhang X, Dong B, et al (2011) Seven novel mutations in the long isoform of the USH2A gene in Chinese families with nonsyndromic retinitis pigmentosa and Usher syndrome Type II. Mol Vis 17: 1537–1552. 21686329 PMC3115748

[8] Sandberg MA, Rosner B, Weigel-DiFranco C, McGee TL, Dryja TP, et al (2008) Disease course in patients with autosomal recessive retinitis pigmentosa due to the USH2A gene. Invest Ophthalmol Vis Sci 49: 5532–5539. 18641288 10.1167/iovs.08-2009 PMC2588642

[9] Ahmed ZM, Smith TN, Riazuddin S, Makishima T, Ghosh M, et al (2002) Nonsyndromic recessive deafness DFNB18 and Usher syndrome type IC are allelic mutations of USHIC. Hum Genet 110: 527–531. 12107438 10.1007/s00439-002-0732-4

[10] Weil D, Kussel P, Blanchard S, Levy G, Levi-Acobas F, et al (1997) The autosomal recessive isolated deafness, DFNB2, and the Usher 1B syndrome are allelic defects of the myosin-VIIA gene. Nat Genet 16: 191–193. 9171833 10.1038/ng0697-191

[11] Auslender N, Sharon D, Abbasi AH, Garzozi HJ, Banin E, et al (2007) A common founder mutation of CERKL underlies autosomal recessive retinal degeneration with early macular involvement among Yemenite Jews. Invest Ophthalmol Vis Sci 48: 5431–5438. 18055789 10.1167/iovs.07-0736

[12] Beit-Ya'acov A, Mizrahi-Meissonnier L, Obolensky A, Landau C, Blumenfeld A, et al (2007) Homozygosity for a novel ABCA4 founder splicing mutation is associated with progressive and severe Stargardt-like disease. Invest Ophthalmol Vis Sci 48: 4308–4314. 17724221 10.1167/iovs.07-0244

[13] Clark JG (1981) Uses and abuses of hearing loss classification. ASHA 23: 493–500. 7052898

[14] Engdahl B, Tambs K (2002) Otoacoustic emissions in the general adult population of Nord-Trondelag, Norway: II. Effects of noise, head injuries, and ear infections. Int J Audiol 41: 78–87. 12467373 10.3109/14992020209101315

[15] Rozen S, Skaletsky HJ (2000) Primer3 on the WWW for general users and for biologist programmers. In: Krawetz S, Misener S, editors. Bioinformatics Methods and Protocols: Methods in Molecular Biology. Totowa, NJ: Humana Press. pp. 365–386. 10.1385/1-59259-192-2:365 10547847

[16] Scanlan MJ, Williamson B, Jungbluth A, Stockert E, Arden KC, et al (1999) Isoforms of the human PDZ-73 protein exhibit differential tissue expression. Biochim Biophys Acta 1445: 39–52. 10209257 10.1016/s0167-4781(99)00033-0

[17] Grillet N, Xiong W, Reynolds A, Kazmierczak P, Sato T, et al (2009) Harmonin mutations cause mechanotransduction defects in cochlear hair cells. Neuron 62: 375–387. 19447093 10.1016/j.neuron.2009.04.006 PMC2691393

[18] Lillo C, Kitamoto J, Williams DS (2006) Roles and interactions of usher 1 proteins in the outer retina. Adv Exp Med Biol 572: 341–348. 17249594 10.1007/0-387-32442-9_48

[19] Bitner-Glindzicz M, Lindley KJ, Rutland P, Blaydon D, Smith VV, et al (2000) A recessive contiguous gene deletion causing infantile hyperinsulinism, enteropathy and deafness identifies the Usher type 1C gene. NatGenet 26: 56–60. 10.1038/79178 10973248

[20] Verpy E, Leibovici M, Zwaenepoel I, Liu XZ, Gal A, et al (2000) A defect in harmonin, a PDZ domain-containing protein expressed in the inner ear sensory hair cells, underlies Usher syndrome type 1C. NatGenet 26: 51–55. 10.1038/79171 10973247

[21] Ouyang XM, Xia XJ, Verpy E, Du LL, Pandya A, et al (2002) Mutations in the alternatively spliced exons of USH1C cause non-syndromic recessive deafness. Hum Genet 111: 26–30. 12136232 10.1007/s00439-002-0736-0

[22] Saihan Z, Stabej Ple Q, Robson AG, Rangesh N, Holder GE, et al (2011) Mutations in the USH1C gene associated with sector retinitis pigmentosa and hearing loss. Retina 31: 1708–1716. 21487335 10.1097/IAE.0b013e31820d3fd1

[23] Roux AF, Faugere V, Le Guedard S, Pallares-Ruiz N, Vielle A, et al (2006) Survey of the frequency of USH1 gene mutations in a cohort of Usher patients shows the importance of cadherin 23 and protocadherin 15 genes and establishes a detection rate of above 90%. J Med Genet 43: 763–768. 16679490 10.1136/jmg.2006.041954 PMC2564578

