# Supplementary material for: Exome Sequencing Identifies a Founder Frameshift Mutation in an Alternative Exon of USH1C as the Cause of Autosomal Recessive Retinitis Pigmentosa with Late-Onset Hearing Loss
Source: PLoS One. 2012 Dec 12;7(12):e51566. doi: 10.1371/journal.pone.0051566 (PMC3520954; doi:10.1371/journal.pone.0051566)

**Figure S1:** **Goldmann perimetry of two patients who are homozygous for the *USH1C* c.1220delG mutation.** A. A 20 year-old patient (MOL0125 II:4) presented in 2007 with severely restricted visual fields which became worse 4 years later. B. The visual field of a 25 year-old patient (MOL0125 II:2) was markedly reduced in 2006 with additional progression 3 years later. The visual fields represent an average of both eyes.


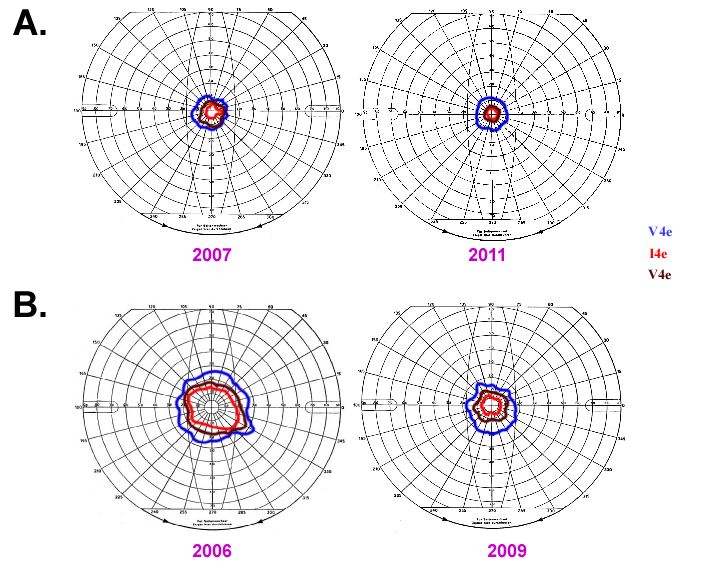

Supplement: Figure S1 — Goldmann perimetry of two patients who are homozygous for the USH1C c.1220delG mutation. A. A 20 year-old patient (MOL0125 II:4) presented in 2007 with severely restricted visual fields which became worse 4 years later. B. The visual field of a 25 year-old patient (MOL0125 II:2) was markedly reduced in 2006 with additional progression 3 years later. The visual fields represent an average of both eyes. (DOCX) [file pone.0051566.s001.docx]
