# Supplementary material for: Exome Sequencing Identifies a Founder Frameshift Mutation in an Alternative Exon of USH1C as the Cause of Autosomal Recessive Retinitis Pigmentosa with Late-Onset Hearing Loss
Source: PLoS One. 2012 Dec 12;7(12):e51566. doi: 10.1371/journal.pone.0051566 (PMC3520954; doi:10.1371/journal.pone.0051566)
Supplement: Table S1 — Primer sequences used to amplify the USH1C exon 15 from gDNA samples and the various transcripts of USH1C. (DOCX) [file pone.0051566.s002.docx]

**Table S1: Primer sequences used to amplify the *USH1C* exon 15 from gDNA samples and the various transcripts of *USH1C***

| **Exon number** | **Forward/ reverse** | **Sequence 5'-3'** |
| --- | --- | --- |
| Primers for gDNA amplification | | |
| 15F | Forward | GGAGGAAGCTGATTACAGGTC |
| 15R | Reverse | CACGGAGGAGCAGGGAGAGCAG |
| Primers for cDNA amplification | | |
| 14F | Forward | ACTGGGGCTCAAAGGAACAGCT |
| 16R | Reverse | TTCCGGATGGTTGGGAATTTGCC |
| 23R | Reverse | TCTTGATGCGTAGGAGCCGGAC |

The melting temperature (Tm) for PCR using primers 15F and 15R was 54^0^C and the PCR product was 180 bp long. Tm for cDNA amplification was 59^0^C and the PCR product size was 189 bp for 14F and 16R and 249 bp for 14F and 23R.
