# Supplementary material for: Exome Sequencing Identifies a Founder Frameshift Mutation in an Alternative Exon of USH1C as the Cause of Autosomal Recessive Retinitis Pigmentosa with Late-Onset Hearing Loss
Source: PLoS One. 2012 Dec 12;7(12):e51566. doi: 10.1371/journal.pone.0051566 (PMC3520954; doi:10.1371/journal.pone.0051566)
Supplement: Table S2 — Homozygosity mapping analysis of arRP families. (DOCX) [file pone.0051566.s003.docx]

**Table S2:** **Homozygosity mapping analysis of arRP families**

| **Family** | **No. of affected individuals** | **Level of consanguinity** | **SNP array**  **used** | **Number of patients tested by SNP array** | **Chromosome** | **Size (range in Mb) of homozygous region** |
| --- | --- | --- | --- | --- | --- | --- |
| MOL0125 | 4 | None | Affy 6.0 | 4 | 11* | 0.905  (16.637 – 17.542) |
| MOL0486 | 2 | 2:2 | Affy 10K | 2 | 1 | 10.816  (204.334 – 215.150) |
|  |  |  |  |  | 11* | 38.98  (6.727-45.707) |

* The *USH1C* mutation is located on chromosome 11 at 17,539,014 (hg19).

Whole-genome SNP data were analyzed by HomozygosityMapper (<http://www.homozygositymapper.org>). In the consanguineous family MOL0486 a homozygous region was defined as a genomic region of over 10 Mb in which all markers were homozygous. No-call SNPs were excluded from the analysis.
