# Supplementary material for: Exome Sequencing Identifies a Founder Frameshift Mutation in an Alternative Exon of USH1C as the Cause of Autosomal Recessive Retinitis Pigmentosa with Late-Onset Hearing Loss
Source: PLoS One. 2012 Dec 12;7(12):e51566. doi: 10.1371/journal.pone.0051566 (PMC3520954; doi:10.1371/journal.pone.0051566)
Supplement: Table S3 — Summary of original exome sequencing data. (DOCX) [file pone.0051566.s004.docx]

**Table S3: Summary of original exome sequencing data**

| **Patient number** | **Total bases produced (Gbp)** | **Mapped reads (%)** | **Number of covered bases (Mbp)** | **Coverage*** |
| --- | --- | --- | --- | --- |
| MOL0125  II:4 | 4.51 | 40.91 Mbp (92%) | 964.89 | 31.17 |
| MOL0486  II:1 | 4.77 | 42.21 Mbp  (88%) | 933.93 | 30.17 |

* The average number of reads per covered nucleotide.
