# Supplementary material for: Exome Sequencing Identifies a Founder Frameshift Mutation in an Alternative Exon of USH1C as the Cause of Autosomal Recessive Retinitis Pigmentosa with Late-Onset Hearing Loss
Source: PLoS One. 2012 Dec 12;7(12):e51566. doi: 10.1371/journal.pone.0051566 (PMC3520954; doi:10.1371/journal.pone.0051566)
Supplement: Table S4 — Statistical analysis of the c.1220delG mutation frequency. (DOCX) [file pone.0051566.s005.docx]

**Table S4: Statistical analysis of the c.1220delG mutation frequency**

|  | **Patients** | | **Controls** | |
| --- | --- | --- | --- | --- |
|  | **Mutant** | **wt** | **Mutant** | **wt** |

| **Chromosome *count** | **16** | **58** | **1** | **237** |
| --- | --- | --- | --- | --- |

| **Genotype** | **M/M** | **M/+** | **+/+** | **M/M** | **M/+** | **+/+** |
| --- | --- | --- | --- | --- | --- | --- |
| **Genotype **count** | **8** | **0** | **29** | **0** | **1** | **118** |

*- The numbers represent the allele count of each studied chromosome in patients versus controls.

**- The numbers represent the different individuals sharing the same genotype of *USH1C*.

P < 0.001 was obtained for both chromosome and genotype count.
