# Supplementary material for: Exome Sequencing Identifies a Founder Frameshift Mutation in an Alternative Exon of USH1C as the Cause of Autosomal Recessive Retinitis Pigmentosa with Late-Onset Hearing Loss
Source: PLoS One. 2012 Dec 12;7(12):e51566. doi: 10.1371/journal.pone.0051566 (PMC3520954; doi:10.1371/journal.pone.0051566)
Supplement: Table S5 — Ocular data of patients with the c.1220delG USH1C gene mutation. (DOCX) [file pone.0051566.s006.docx]

**Table S5: Ocular data of patients with the c.1220delG *USH1C* gene mutation**

| **Patient number** | **Patient age (years)** | **Visual acuity** | **Refraction** | **Full field ERG results** | | | **Color vision** |
| --- | --- | --- | --- | --- | --- | --- | --- |
|  |  |  |  | **Averaged mixed cone-rod response (μV)** | **Averaged cone flicker - 30Hz (IT)** | **Rod response - blue light (b, μV)** |  |
| MOL0486 II:3 | 13 | 1.0- | +4.25 | Undetectable | Trace response | Undetectable | NA |
| MOL0486 II:1 | 19 | 1.0- | -3.75 | a: 67  b: 103 | 7 (38.0) | 76 | NA |
| MOL0125 II:4 | 20 | 0.80 | 0.00 | Undetectable | 10.8 (44.5) | Undetectable | NA |
| MOL0125 II:3 | 22 | 0.5 | NA | Undetectable | Undetectable | Undetectable | NA |
| MOL0125 II:2 | 25 | 0.625 | +1.00 | a: 49  b: 96 | 20.2 (32.3) | 52 | BE Within Normal Range |
| MOL0125 II:1 | 27 | 0.5 | -1.00 | a: 27  b: 31 | 14.2 (33.5) | BE≈ 10 | BE Within Normal Range |
| TB16/R12 | 33 | 0.4- | -1.50 | Undetectable | 6 (38.0) | BE: 0 | NA |
| MOL0798-1 | 38 | 0.2 | -8.00 | Undetectable | Undetectable | Undetectable | NA |
| MOL0887-1 | 40 | 0.7 | +2.00 | NA | NA | NA | NA |
| MOL0887-2 | 41 | 0.6 | -1.75 | Undetectable | Undetectable | Undetectable | Undetectable |
| MOL0887-3 | 44 | 0.7 | -1.00 | NA | NA | NA | NA |
| MOL1023-1 | 72 | 0.24 | NA | Undetectable | NA | NA | NA |

NA- data not available. BE- both eyes. Visual acuity is presented as mean spherical equivalent of both eyes.

Full field electroretinograms including the following: Dark-adapted mixed cone-rod a and b-wave amplitude (in μV, normal a-wave 90-350 μV, normal b-wave 380-630 μV); Light-adapted cone flicker amplitude (in μV, normal 60-144 μV) and implicit time (IT, in msec, normal 27-33 msec), and dark-adapted rod response b-wave amplitude (in μV, normal range 200-500 μV).
