# Supplementary material for: Exome Sequencing Identifies a Founder Frameshift Mutation in an Alternative Exon of USH1C as the Cause of Autosomal Recessive Retinitis Pigmentosa with Late-Onset Hearing Loss
Source: PLoS One. 2012 Dec 12;7(12):e51566. doi: 10.1371/journal.pone.0051566 (PMC3520954; doi:10.1371/journal.pone.0051566)
Supplement: Table S6 — Hearing function tests: Audiometry and Transient Evoked Otoacoustic Emissions (TEOAEs). (DOCX) [file pone.0051566.s007.docx]

**Table S6: Hearing function tests: Audiometry and Transient Evoked Otoacoustic Emissions (TEOAEs)**

| **Patient number** | **Patient age (years)** | **Audiometry** | **Severity** | **Audiogram**  **type** | **TEOA's** |
| --- | --- | --- | --- | --- | --- |
| MOL0486 II:3 | 13 | Both ears WNL | - | - | Both ears WNL |
| MOL0486 II:1 | 19 | Both ears WNL | - | - | Both ears WNL |
| MOL0125 II:4 | 20 | Both ears WNL | - | - | Both ears WNL |
| MOL0125 II:2 | 25 | Both ears WNL | - | - | Both ears WNL |
| TB16/R12 | 33 | Both ears WNL | - | - | Both ears WNL |
| MOL0798-1 | 38 | Both ears SNHL | Mild to Moderate | High tone (8-12KHz) | Both ears WNL |
| MOL0887-1 | 40 | Both ears WNL | - | - | Both ears WNL |
| MOL0887-2 | 41 | Both ears SNHL | Mild | High tone (4-8KHz) | NA |
| MOL0887-3 | 44 | Both ears SNHL | Severe | Down-slopping | NA |
| MOL1023-1 | 72 | Both ears SNHL * | Moderate to severe | Down-slopping | No Response |

SNHL - Sensorineural hearing loss. WNL - Within normal limits. TEOAE - Transient evoked otoacoustic emission. NA- data not available.

* Probably combined presbyacusis and retinitis pigmentosa is related to the *USH1C* genotype (homozygosity for the c.1220delG mutation).
