# Supplementary material for: Exome Sequencing Identifies a Founder Frameshift Mutation in an Alternative Exon of USH1C as the Cause of Autosomal Recessive Retinitis Pigmentosa with Late-Onset Hearing Loss
Source: PLoS One. 2012 Dec 12;7(12):e51566. doi: 10.1371/journal.pone.0051566 (PMC3520954; doi:10.1371/journal.pone.0051566)
Supplement: Table S7 — Splice-variants produced by the human USH1C gene. (DOCX) [file pone.0051566.s008.docx]

**Table S7: Splice-variants produced by the human *USH1C* gene**

| **Splicing variant** | **Exons included** | **Expression pattern** | **Previous variant name** |
| --- | --- | --- | --- |
| USH1C_v1 | 1-14,16-28 |  | b3 (NCBI) |
| USH1C_v2 | 1-15,22-26,28 | Ubiquitous | a (NCBI)  PDZ-73 (Scanlan et al. 1999) |
| USH1C_v3 | 1-14,22-26,28 |  | PDZ-45 (Scanlan et al. 1999) |
| USH1C_v4 | 1-28 | Ubiquitous | Not reported previously |

The sequence of variants USH1C_v2 and USH1C_v4 was verified by sequencing of RT-PCR products.
